# Pre-Peritoneal Dialysis Peritonitis After Saline Infusion Sonohysterogram in a Patient With an Embedded Catheter: A Case Report

**DOI:** 10.1177/20543581231156854

**Published:** 2023-02-17

**Authors:** Alexandre Cosmatos, Brendan McCormick, Brian Blew, Pierre Antoine Brown

**Affiliations:** 1 Faculty of Medicine, University of Ottawa, ON, Canada; 2 Division of Nephrology, Department of Medicine, University of Ottawa, ON, Canada; 3 Kidney Research Centre, Ottawa Hospital Research Institute, ON, Canada; 4 Division of Urology, Department of Surgery, The Ottawa Hospital, University of Ottawa, ON, Canada

**Keywords:** peritonitis, peritoneal dialysis, embedded peritoneal dialysis catheter, antibiotic prophylaxis, pre-peritoneal dialysis peritonitis

## Abstract

**Rationale:**

Clear guidelines currently exist regarding antibiotic prophylaxis for patients on peritoneal dialysis (PD) prior to common diagnostic procedures. However, these guidelines do not include patients with subcutaneously embedded PD catheters who are awaiting PD initiation although both these populations share a great deal of risk factors for infections. Issues regarding antibiotic prophylaxis and avoidable infections are bound to keep occurring if physicians are not conscious of the risks of infections shared by all patients suffering from renal failure.

**Presenting concerns:**

Two weeks after a saline infusion sonohysterography (SIS), a 48-year-old woman with chronic kidney disease (CKD) G5 ND, type 2 diabetes, a subcutaneously embedded PD catheter, and prior abnormal uterine bleeding presented to the emergency department complaining of nausea, vomiting, diarrhea, weakness, and abdominal pain. The patient received no antibiotic prophylaxis prior to her SIS.

**Diagnoses:**

The final diagnosis of peritonitis was established after acute kidney injury, gastroenteritis, and small bowel obstruction were considered and ruled out. A delay in the final diagnosis occurred because of the complex presentation, the fact that the patient had not yet initiated PD, and the presence of concomitant anion gap metabolic acidosis and an acute elevation of the patient’s creatinine.

**Interventions:**

The patient was started on broad-spectrum intravenous antibiotics when the diagnosis of peritonitis was established. Insulin and intravenous bicarbonate infusions were used to correct the patient’s anion gap metabolic acidosis. Surgical debridement of the necrotic subcutaneous tissue and removal of the embedded PD catheter were necessary.

**Outcomes:**

The patient’s infection resolved completely as did her anion gap metabolic acidosis. The patient had to transfer permanently from PD to hemodialysis for her renal replacement therapy.

**Teaching points:**

This case report serves as a good reminder that physicians should keep in mind the possibility of peritonitis in patients with embedded PD catheters. As these patients are also at risk of infections, antibiotic prophylaxis should be used in patients with embedded catheters in the same way it is used for PD patients prior to obstetrical, gynecological, or gastrointestinal procedures.

## Abrégé

**Justification:**

Il existe des directives claires quant à la prophylaxie antibiotique à utiliser préalablement aux procédures de diagnostic courantes chez les patients sous dialyse péritonéale (DP). Les patients disposant d’un cathéter de DP implanté sous-cutané en attendant le début de la dialyse ne sont pas inclus dans ces recommandations, même si cette population partage plusieurs facteurs de risque d’infections avec les patients déjà sous DP. Des enjeux liés à la prophylaxie antibiotique et aux infections évitables continueront de se poser si les médecins ignorent les risques d’infections partagés par tous les patients souffrant d’insuffisance rénale.

**Présentation du cas:**

Une femme âgée de 48 ans atteinte d’insuffisance rénale chronique (IRC) G5 ND et de diabète de type 2 s’étant présentée aux urgences deux semaines après une sono-hystérographie (SHG) avec infusion intra-utérine de solution saline. La patiente portait un cathéter de PD implanté sous-cutané et avait déjà eu des saignements utérins anormaux dans le passé. Elle se plaignait de nausées, de vomissements, de diarrhées, de faiblesse générale et de douleurs abdominales. Elle n’avait reçu aucune prophylaxie antibiotique avant la SHG.

**Diagnostic:**

Le diagnostic final de péritonite a été établi après que l’insuffisance rénale aiguë, la gastro-entérite et une obstruction de l’intestin grêle aient été envisagées et écartées. Le diagnostic final a été retardé en raison de la présentation complexe, du fait que la patiente n’avait pas encore amorcé la DP et de la présence concomitante d’une acidose métabolique à trou anionique et d’une élévation subite de la créatinine.

**Interventions:**

La patiente a reçu des antibiotiques à large specter par voie intraveineuse lorsque le diagnostic de péritonite a été établi. Des infusions d’insuline et de bicarbonate par voie intraveineuse ont été utilisées pour corriger l’acidose métabolique à trou anionique. Un débridement chirurgical des tissus sous-cutanés nécrosés et l’ablation du cathéter PD se sont avérés nécessaires.

**Résultats:**

L’infection a guéri complètement, tout comme l’acidose métabolique à trou anionique. La patiente a dû passer définitivement de la DP à l’hémodialyse pour son traitement de suppléance rénale.

**Enseignements tirés:**

Ce cas illustre bien que les médecins devraient toujours garder les risques de péritonite à l’esprit lorsqu’ils traitent des patients portant un cathéter de PD implanté sous-cutané. Puisque ces patients présentent eux aussi un risque d’infection, la prophylaxie antibiotique devrait leur être administrée avant les procédures obstétricales, gynécologiques ou gastro-intestinales, comme c’est le cas pour les patients sous DP.

## Introduction

Patients suffering from chronic kidney disease (CKD) are known to be at increased risk of infections because impaired kidney function alters normal immune system function.^1-3^ This relative immunocompromised state among peritoneal dialysis (PD) patients may explain why PD-associated bacterial peritonitis remains one of the most common complications of PD and a major cause of renal replacement therapy (RRT) modality change from PD to hemodialysis (HD).^4-6^ Peritoneal dialysis–related peritonitis occurs mainly through these major routes: periluminal contamination, transluminal contamination, hematological spread after medical procedures, and bacterial translocation to the peritoneum after certain diagnostic procedures.^7-10^

Contamination after a diagnostic procedure, whether hematological or through translocation to the peritoneum, is a well-known risk factor for PD peritonitis, and guidelines established by the International Society of Peritoneal Dialysis (ISPD) recommend peri-procedure antibiotic prophylaxis for patients on PD undergoing obstetrical, gynecological, or gastrointestinal procedures.^4,11^ The peri-procedure prophylaxis section in the ISPD guidelines does not include recommendations for patients who have a subcutaneously embedded PD catheter but have not yet initiated dialysis.^4^

Subcutaneously embedded PD catheters are used in some centers as insertion of the catheter weeks to months in advance of the need for dialysis helps with planification of care for patients who will need PD in the future and because this technique allows for healing of the cuff in a sterile environment.^9^ As an embedded PD catheter is covered by the patient’s skin, it exists in a sterile environment and makes transluminal or periluminal contamination from exit-site bacteria very unlikely.^9^ However, hematogenous spread or translocation of bacteria is still possible. Herein, we present the first reported case of a patient with an embedded PD catheter who developed a catheter infection following a saline infusion sonohysterography (SIS).

### Presenting Concerns

A 48-year-old woman with CKD G5 ND secondary to diabetic nephropathy had a subcutaneously embedded PD catheter inserted 2 months prior to her emergency room (ER) visit, in anticipation of requiring dialysis within 6 months. In the interim, she developed abnormal uterine bleeding (AUB) which was investigated with SIS without prophylactic antibiotics. Almost immediately after, she developed crampy lower abdominal pain which persisted for days and then was followed by nausea, vomiting, diarrhea, and weakness. She then presented to the ER for further evaluation on day 0 (Table 1).

**Table 1.** Timeline of Clinical Events.

Date	Clinical event
Day −28	Seen in the Multi Care Kidney clinic with worsening renal function and already embedded PD catheter PD catheter training scheduled for the following month
Day −13	SIS for AUB with symptoms of abdominal pain and diarrhea soon after. No prophylactic antibiotics were given before the procedure
Day 0	Sent to ER for evaluation: Symptoms attributed initially to acute on chronic kidney injury and viral gastroenteritis. Patient admitted for PD start and nephrologist consulted for PD catheter exteriorization
Day 1	CT findings of possible necrotizing abdominal wall infection. Intravenous antibiotics are initiated
Day 2	Surgical debridement and PD catheter removal. Hemodialysis initiated with Vascular Catheter
Day 14	Discharged permanently on HD

*Note.* This table includes the complete relevant timeline of clinical events, starting before the patient’s presentation to the ER and ending with the conclusion of the case. PD = peritoneal dialysis; SIS = saline infusion sonohysterography; AUB = abnormal uterine bleeding; ER = emergency room; CT = Computed tomography; HD = hemodialysis.

## Clinical Findings

Upon presentation to the ER, the patient was afebrile, had a blood pressure of 122/71 mm Hg, a heart rate of 85 beats per minute, and an oxygen saturation of 96% in ambient air. Physical examination revealed abdominal wall edema and diffuse tenderness without any signs of a tunnel infection, such as erythema, tenderness, or swelling along the area over the catheter.^11^ Rebound tenderness was not assessed by the medical team during physical examination.

## Diagnostic Focus and Assessment

Initial laboratory investigations showed the following results—white blood cells: 17.0 × 10^9^/L, hemoglobin: 80 g/L, sodium: 121 mmol/L, potassium: 5.7 mmol/L, and creatinine: 1121 µmol/L. The patient’s serum creatinine level was 583 µmol/L 1 month prior.

Laboratory analyses also demonstrated concomitant anion gap metabolic acidosis (AGMA) with a pH of 7.24, glucose of 20 mmol/L, and serum bicarbonate of 14 mEq/L. A stool sample done in the context of diarrhea and abdominal pain later returned positive for *Clostridium difficile* toxins.

**Figure 1.**
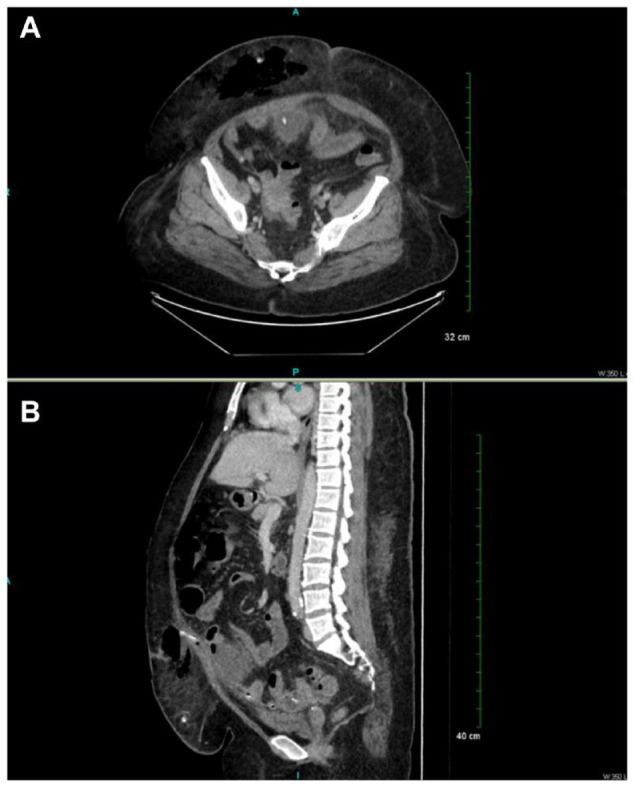
Abdominal computed tomography (CT) scan without contrast. (A) CT scan of the abdomen (axial view) demonstrates extensive organized subcutaneous and rectus muscle edema with extensive gas in the abdominal wall. (B) CT scan of the abdomen (sagittal view) demonstrates rim-enhancing air-fluid levels in mildly dilated and thickened small bowel, with mesenteric edema and fluid throughout the colon.

An abdominal radiograph confirmed that the catheter was in good position, but it also showed dilated small bowel loops, suggesting potential ileus or obstruction.^12^ Computed tomography (CT) imaging showed extensive air in the abdominal wall, subcutaneous edema, stranding, and skin thickening in addition to a rim-enhancing air and fluid collection in the abdominal wall inseparable from the catheter thought to be an abscess (Figure 1). The adjacent rectus abdominis muscle was thickened and edematous as well (Figure 1). There was also suggestion of enterocolitis with associated ileus (Figure 1).

## Therapeutic Focus and Assessment

Empirical piperacillin-tazobactam was initiated after the CT findings of a necrotizing abdominal infection. Insulin and intravenous bicarbonate infusions were used to correct the patient’s AGMA. The patient underwent urgent surgical removal of the subcutaneously embedded PD catheter and necrotic subcutaneous fat as well as surgical debridement of her right abdominal wall abscess. The operative findings revealed a large peri-catheter subcutaneous abscess. The PD catheter was already very loose from the abdominal fascia and removed without dissection. The subcutaneous abscess cavity did not extend within the peritoneal cavity. A temporary vascular HD catheter was inserted. Abscess fluid samples were sent for culture and grew *Peptostreptococcus indolicus*.

## Follow-up and Outcomes

The surgery was unremarkable, apart from brief intraoperative hypotension requiring intermittent vasopressors. A Penrose drain was also installed to assure complete drainage of the abscess. The patient was transitioned permanently to HD and a tunneled central venous catheter was inserted prior to discharge. Postoperatively, a regimen consisting of piperacillin/tazobactam, vancomycin, and clindamycin led to the complete resolution of the patient’s peritonitis. The patient was discharged and continues outpatient HD.

## Discussion

We present the first reported case of peritonitis in a patient with an embedded PD catheter not yet on dialysis after undergoing an elective SIS. While the risks of developing peritonitis after invasive procedures for PD patients are well understood,^4^ a gap in the literature exists regarding these risks for patients with embedded PD catheters not yet on dialysis.

The ISPD has clear recommendations to give prophylactic antibiotics to patients on PD for obstetric, gynecological, or gastrointestinal procedures to prevent peritonitis.^4,13,14^ However, no recommendations exist in the literature regarding patients with embedded PD catheters. Consequently, we suggest that these patients receive the same antibiotic prophylaxis prior to gastrointestinal, obstetric, and gynecological procedures than patients on PD.

The ISPD guidelines for PD-related peritonitis also establish a clear definition for the diagnosis of PD-associated peritonitis.^4^ The diagnosis is based on a combination of 2 or more of the following: (1) consistent clinical features such as abdominal pain and/or cloudy effluent, (2) elevated leukocyte count in the dialysis effluent, and (3) a positive dialysis effluent culture.^4^

While this definition is sufficient for most instances of PD-associated peritonitis, many factors led to challenges in diagnosis in this case. First, the presence of a subcutaneously embedded catheter instead of an exteriorized one and the fact that the patient was yet to initiate PD likely delayed the clinical suspicion of peritonitis. Second, in the absence of regular PD exchanges, analysis of the effluent was impossible, which is a component of PD-related peritonitis diagnosis.^4,5^ Third, there were no common signs of PD-associated peritonitis, other than diffuse abdominal pain.^4,5^ Fourth, the presence of a foreign body itself, such as a PD catheter, represents a heightened risk for infection, as it can act as a support for bacterial biofilm.^15^ The presence of an embedded catheter coupled with altered immune function due to CKD makes it more likely to develop peritonitis following peritoneal seeding. Finally, cultures could only be taken at the time of surgical intervention, further delaying the final diagnosis of *Peptostreptococcus indolicus* peritonitis.

*Peptostreptococcus indolicus* is a gram-positive anaerobic coccus normally found as a colonizing organism of human mucocutaneous flora in the mouth, the gastrointestinal tract, and the female genitourinary tract.^16,17^ Gram-positive anaerobic cocci are frequently isolated in infections caused by obstetrical or gynecological procedures.^16,17^

Intraabdominal infection following SIS is rare, with an incidence of less than 1%.^18,19^ In addition, it is exceedingly unusual for these to require surgical intervention like our patient did.^18,19^ Therefore, the isolated pathogen, the contiguous peri-catheter abscess formation along the subcutaneous portion of the PD catheter, and the temporal relationship between the procedure and the infection in the context of a lack of antibiotic prophylaxis prior to the SIS are highly suggestive that the patient’s infection developed because of the preceding SIS. Thus, in this case, it is likely that the embedded PD catheter played a role as a potentiating agent for a severe infection.

This case highlights the importance for clinicians to keep peritonitis in the differential diagnosis of vague abdominal pain in patients with a PD catheter in situ even if they have not yet initiated PD. It also illustrates the importance of appropriate abdominal imaging in similar complex cases. As the lack of prophylactic antibiotics at the time of an elective gynecological procedure likely led to the severe infection, we emphasize the following takeaway points for the reader:

1. Patients who have an existing PD catheter, including subcutaneously embedded ones, are at risk of peritonitis even if PD has not yet been initiated.^1-3^
2. Physicians should be aware of the risk of peritonitis due to the presence of a PD catheter alone and consider this diagnosis even in the context of vague abdominal pain.
3. Though not stated explicitly in the ISPD guidelines, antibiotic prophylaxis should apply to patients with embedded or exteriorized PD catheters, regardless of the dialysis status. Routine antibiotic prophylaxis should therefore be administered prior to invasive procedures such as colonoscopies or gynecological procedures to prevent PD-associated peritonitis.^4,7,10,14^
